# Knee osteoarthritis management across the joint-preservation continuum: current evidence and future directions

**DOI:** 10.3389/fsurg.2026.1924870

**Published:** 2026-09-28

**Authors:** Joshua Kulpinski, Andrew Carusi, Michael Langworthy

**Affiliations:** Southcoast Health, Dartmouth, MA, United States

**Keywords:** biomarkers, disease-modifying osteoarthritis drugs, intra-articular therapy, joint preservation, knee, osteoarthritis

## Abstract

Knee osteoarthritis is a leading cause of chronic pain, disability, and mobility limitation, with increasing prevalence driven by population aging, obesity, and joint injury. Although historically viewed as passive cartilage “wear and tear,” knee osteoarthritis is now understood as a heterogeneous whole-joint disease involving cartilage matrix degradation, chondrocyte dysfunction, synovial inflammation, subchondral bone remodeling, genetic and epigenetic susceptibility, and altered biomechanics. This narrative review examines current and emerging strategies for knee osteoarthritis management across the joint-preservation continuum. PubMed/MEDLINE, the Cochrane Library, and Google Scholar were searched through August 2026, prioritizing systematic reviews, randomized controlled trials, clinical practice guidelines, and relevant primary studies, with selective inclusion of preclinical evidence for emerging therapies. This broader biological understanding has identified therapeutic targets and supports a joint-preservation continuum that extends from weight management, exercise, and pharmacologic symptom control to intra-articular therapies, cartilage restoration, and investigational molecular or gene-based interventions. Currently available intra-articular therapies may provide symptom relief in selected patients but have not demonstrated consistent structural disease modification, and guideline recommendations differ for several intra-articular, orthobiologic, and regenerative treatments because of substantial heterogeneity in the available evidence. Emerging approaches, including cytokine-directed therapy, photobiomodulation, platelet-rich plasma, and tissue engineering, aim to influence disease mechanisms, although their clinical maturity and evidentiary strength vary substantially. Future precision-medicine strategies may integrate clinical phenotype, biological profiles, imaging, biomarkers, and genetic risk to identify patients most likely to progress or respond to specific interventions. At present, no disease-modifying osteoarthritis drug is approved for knee osteoarthritis. Nevertheless, biologically informed, stage-appropriate care may help preserve function, address modifiable disease drivers, and extend native-joint health while maintaining the essential role of total knee arthroplasty for end-stage disease.

## 1 Introduction

Osteoarthritis (OA) affected an estimated 595 million people globally in 2020, and the number of knee osteoarthritis (KOA) cases is projected to increase by approximately 75% by 2050 (1). KOA contributes to pain, stiffness, reduced mobility, work limitation, and potential loss of independence, resulting in substantial direct medical and societal costs (1, 2). Its burden is compounded by longer life expectancy, rising obesity, and the accumulation of traumatic and occupational joint exposures (2). Management has traditionally emphasized pain relief through exercise, weight reduction, nonsteroidal anti-inflammatory drugs (NSAIDs), intra-articular injections, and ultimately total knee arthroplasty (TKA) (2). Although these interventions can improve symptoms and function, most do not stop structural progression of the joint (2). TKA remains highly effective for appropriately selected patients with end-stage disease; however, persistent postoperative symptoms in a subset of patients, revision risk, and the increasing use of arthroplasty in younger populations highlight the rationale for preserving native joint function during the earlier stages of KOA (2).

A major clinical challenge is the variable relationship between symptom duration, symptom severity, and structural disease (2). Patients may have substantial pain despite modest radiographic change, whereas others have advanced radiographic abnormalities with limited symptoms (3, 4). This symptom-structure discordance complicates disease staging and clinical assessment (4, 5). Pain in KOA may also involve overlapping nociceptive, neuropathic-like, and nociplastic components, further contributing to variability between structural findings and symptom severity and supporting a more individualized approach to treatment (6). A biologically informed approach does not replace clinical judgment; rather, it helps explain why the same intervention may benefit one phenotype while offering little value to another (7). Representative anteroposterior and lateral radiographs illustrate the structural contrast between knees classified as Kellgren–Lawrence grades 1 and 3 (Figures 1, 2).

**Figure 1:**
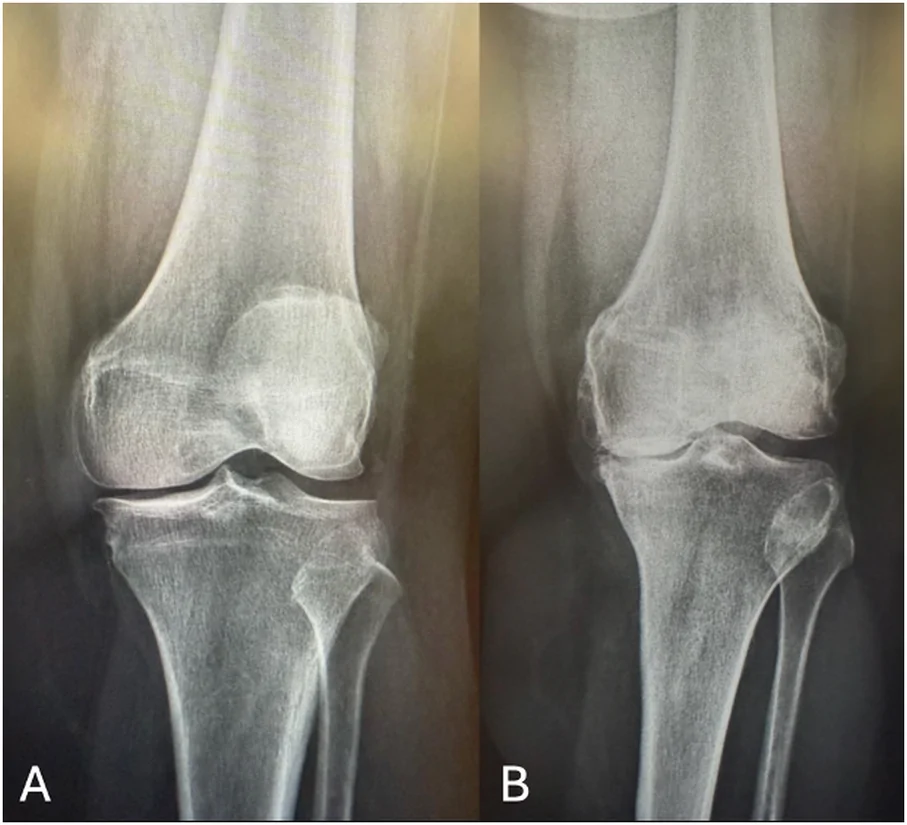
Representative weight-bearing anteroposterior radiographic comparison of knees with Kellgren–Lawrence grades 1 and 3. **(A)** Weight-bearing anteroposterior radiograph classified as Kellgren–Lawrence grade 1, with preserved tibiofemoral joint space and minimal radiographic degenerative change. **(B)** Weight-bearing anteroposterior radiograph classified as Kellgren–Lawrence grade 3, demonstrating definite joint-space narrowing, osteophyte formation, subchondral sclerosis, and degenerative contour changes. The Kellgren–Lawrence grades were assigned by an orthopedic surgeon following retrospective review of the weight-bearing anteroposterior radiographs; no independent or quantitative radiographic assessment was performed. Joint-space narrowing is shown as an indirect radiographic marker of cartilage loss. The images are representative, de-identified examples from different patients and are intended for qualitative visual comparison rather than quantitative measurement or demonstration of disease progression within the same patient. Panel B was mirrored horizontally to standardize orientation for visual comparison. Images are used with patient consent. Courtesy of Dr. Michael Langworthy.

**Figure 2:**
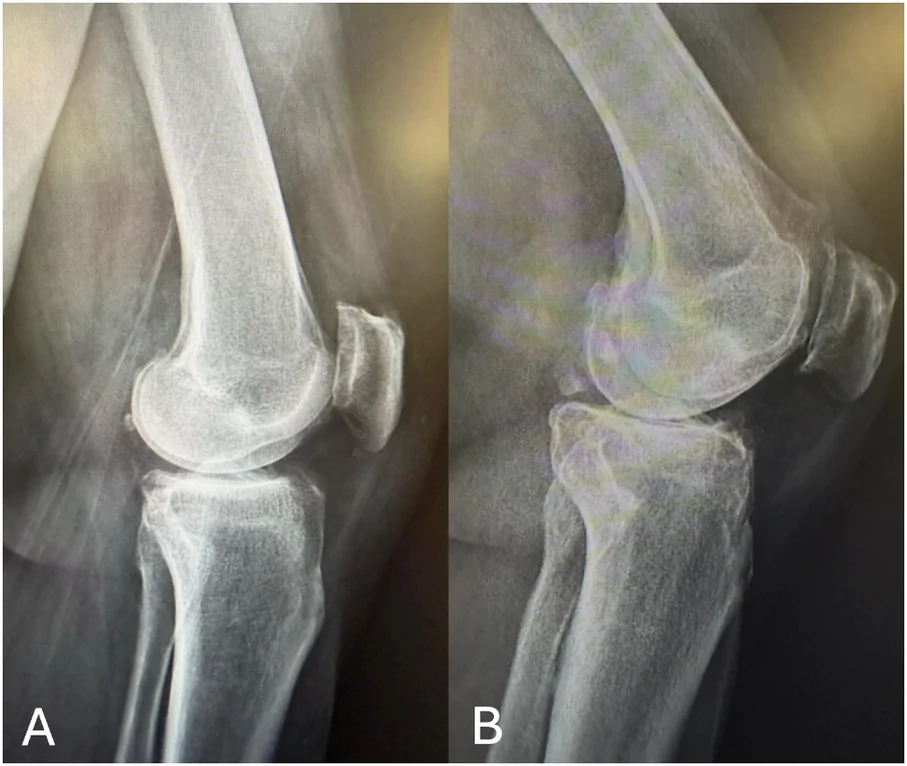
Representative weight-bearing lateral radiographic views corresponding to the knees shown in Figure 1. **(A)** Weight-bearing lateral radiograph of the knee classified as Kellgren–Lawrence grade 1 on the corresponding anteroposterior view, demonstrating preserved joint alignment and minimal degenerative change. **(B)** Weight-bearing lateral radiograph of the knee classified as Kellgren–Lawrence grade 3 on the corresponding anteroposterior view, demonstrating more advanced degenerative change, including osteophyte formation and subchondral sclerosis. Kellgren–Lawrence grading was based on retrospective review of the corresponding weight-bearing anteroposterior radiographs rather than the lateral views. The images represent the same knees shown in Figure 1 from a different radiographic orientation and are intended for qualitative visual comparison rather than quantitative measurement or demonstration of disease progression within the same patient. Panel B was mirrored horizontally to standardize orientation for visual comparison. Images are de-identified and used with patient consent. Courtesy of Dr. Michael Langworthy.

The conceptual model of KOA has therefore changed. Rather than isolated cartilage attrition, KOA is recognized as a disorder of the entire synovial joint, involving articular cartilage, synovium, subchondral bone, menisci, ligaments, joint capsule, and periarticular musculature (8). Abnormal mechanical loading contributes to cartilage degeneration through chondrocyte death, inflammatory signaling, and extracellular-matrix degradation (9), while genetic susceptibility also contributes to the development and progression of primary KOA (10). This narrative review focuses on adult knee osteoarthritis, including both primary and post-traumatic disease, and examines how advances in disease biology inform contemporary clinical management, joint-preservation strategies, and emerging mechanism-based and regenerative therapies. “Joint preservation” is used broadly to describe strategies that maintain native-joint function or tissue; it does not imply that every intervention is a proven structural disease modifier.

### 1.1 Literature search and review approach

A narrative literature search was conducted using PubMed/MEDLINE, the Cochrane Library, and Google Scholar, with the final search performed in August 2026. Search concepts included knee osteoarthritis, disease pathophysiology, cartilage and subchondral bone biology, inflammation, joint preservation, intra-articular therapies, orthobiologics, regenerative approaches, and disease-modifying osteoarthritis drugs. Reference lists of relevant reviews and primary studies were also examined to identify additional literature. No formal language or publication-date restrictions were applied.

Priority was given to systematic reviews and meta-analyses, randomized controlled trials, long-term follow-up studies, clinical practice guidelines, and regulatory or product-labeling information when applicable. Preclinical studies were included selectively when necessary to describe emerging biological mechanisms or investigational therapies for which clinical evidence remains limited. When multiple studies were available for the same treatment or topic, priority was given to studies based on the strength and relevance of the evidence, methodological quality, recency, duration of follow-up, and relevance to the scope of this review. The primary literature search and study selection were conducted by the lead author, with a small number of additional relevant studies identified by a coauthor. The selected literature and its interpretation were reviewed and discussed among the authors, with any differences resolved through discussion and consensus. Study selection was not performed through independent duplicate screening.

Because this article is a narrative rather than a systematic review, study identification and selection were not performed according to a prespecified systematic review protocol. Therapeutic examples were selected to represent major contemporary treatment categories, differing biological targets, clinically relevant or investigational approaches, and contrasting clinical trial outcomes; therefore, the therapies discussed are illustrative rather than an exhaustive review of all available or investigational treatments.

## 2 Biological discoveries driving therapeutic innovation

### 2.1 Chondrocytes and extracellular-matrix failure

Articular cartilage is an avascular tissue whose resident chondrocytes maintain an extracellular matrix rich in type II collagen and proteoglycans such as aggrecan. This matrix supports joint articulation and distributes mechanical loads, but the tissue's low cellularity and limited regenerative capacity make restoration challenging once substantial damage has occurred (11). In healthy cartilage, chondrocytes balance matrix synthesis and degradation. In KOA, inflammatory and mechanical stimuli shift them toward a catabolic, matrix-degrading phenotype (12).

Matrix metalloproteinase-13 (MMP-13) degrades type II collagen, whereas a disintegrin and metalloproteinase with thrombospondin motifs-4 and -5 (ADAMTS-4 and ADAMTS-5) degrade aggrecan (13). Breakdown of the collagen-proteoglycan network weakens cartilage and contributes to progressive cartilage damage (14). Aging amplifies this imbalance through reduced anabolic responsiveness, oxidative stress, impaired autophagy, apoptosis, and cellular senescence (15–17). Senescent chondrocytes may remain metabolically active and release inflammatory mediators and proteases as part of a senescence-associated secretory phenotype, further impairing matrix homeostasis. These findings have made cellular senescence a potential therapeutic target in KOA. Strategies aimed at selectively eliminating senescent cells (senolytics) or suppressing their pro-inflammatory and matrix-degrading secretory phenotype (senomorphics) are under investigation; however, these strategies remain largely preclinical, and their clinical role is not yet established (17).

### 2.2 Synovium, cytokines, subchondral bone, and pain

Synovitis is common in KOA and can be present in both early and advanced disease (18). Activated synoviocytes, macrophages, and other immune cells produce mediators including interleukin-1β (IL-1β), IL-6, and tumor necrosis factor-α (TNF-α), which can suppress matrix synthesis and increase MMP and ADAMTS expression (19). Cytokine profiles vary with OA stage and activity, and causal evidence differs among individual inflammatory mediators (19, 20).

Subchondral bone also participates actively in OA. Altered loading and remodeling are associated with bone marrow lesions, microdamage, sclerosis, osteophyte formation, and neurovascular changes (21). Biochemical and mechanical interactions across the osteochondral unit can link cartilage loss with bone remodeling (22). Because articular cartilage is aneural, OA pain arises primarily from innervated joint tissues, including the synovium, subchondral bone, joint capsule, and ligaments (21, 23). Peripheral and central sensitization help explain why radiographic severity and pain do not always correspond (24).

### 2.3 Genetics, epigenetics, and molecular heterogeneity

KOA has a polygenic architecture. Genome-wide association studies have identified an expanding set of susceptibility loci involving skeletal development, cartilage biology, joint morphology, and inflammatory regulation (25). Genetic risk generally reflects the cumulative effect of many variants rather than a single causal mutation. Epigenetic processes, including DNA methylation, histone modification, and noncoding-RNA regulation, provide a mechanism through which aging, obesity, inflammation, and mechanical loading may alter gene activity without changing DNA sequence (26, 27).

A recent UK Biobank study partitioned a KOA polygenic risk score into body mass index (BMI)-associated and BMI-independent components. Of 146 KOA-associated variants, approximately half overlapped with BMI loci; the BMI-independent score remained associated with incident KOA after BMI adjustment (28). This study improves biological interpretation but does not yet justify treatment selection. The cohort was limited to White European participants, and clinical use will require broader validation, calibration with conventional risk factors, and evidence that risk-guided intervention improves outcomes.

### 2.4 Mechanical stress and post-traumatic osteoarthritis

Anterior cruciate ligament (ACL) rupture and meniscal injury demonstrate how mechanical and biological mechanisms converge (29, 30). Long-term studies suggest that approximately half of patients with diagnosed ACL or meniscal tears may develop symptomatic or radiographic OA within 10–20 years, despite risk varying with injury pattern, meniscal loss, alignment, obesity, activity, and reinjury (30). Trauma may damage cartilage and subchondral bone, alter contact mechanics, and initiate hemarthrosis and cytokine release (30, 31). Human cartilage obtained after ACL rupture has shown early collagen cleavage resembling established OA, while impact loading of human cartilage samples induces chondrocyte apoptosis and glycosaminoglycan release (32, 33).

ACL reconstruction can restore mechanical stability but does not eliminate the risk of post-traumatic OA (34). Risk-reduction strategies therefore include meniscal preservation, restoration of stability and alignment, and investigation of biologic interventions targeting the post-injury inflammatory environment (34–36).

## 3 The joint-preservation continuum

The modern therapeutic goal is not merely to postpone surgery at any cost. It is to preserve acceptable pain control, mobility, participation, and physical function while matching treatment burden to disease severity and patient priorities (37–39). The Lancet Commission emphasized OA heterogeneity, prevention, and individualized management (40). Because currently available therapies predominantly modify symptoms, interest has expanded toward disease-modifying osteoarthritis drugs (DMOADs) intended to preserve, repair, or regenerate joint tissues (41). No DMOAD is currently approved for clinical use (41).

A useful continuum begins with education, exercise, weight management, and risk-factor modification. Pharmacologic and intra-articular therapies may then support symptom control and rehabilitation (42). In selected patients, mechanical or surgical preservation, including meniscal repair, ligament stabilization, osteotomy, or focal cartilage restoration, may address a correctable structural problem (43–46). New biologic, molecular, and gene-based therapies continue to be developed and evaluated. Arthroplasty is appropriate when pain and functional loss become unacceptable and joint-preserving measures are unlikely to provide sufficient benefit (47). Although clinical trajectories vary substantially, disease stages may overlap, and progression is often nonlinear (48), foundational interventions remain important throughout the disease course (38). This framework also requires precise outcome language. Symptom modification refers to improvement in pain, stiffness, function, or quality of life. Structural modification requires credible evidence that cartilage loss, joint space narrowing, bone remodeling, or another validated tissue endpoint changes over time (49). Regeneration implies restoration of damaged tissue with sufficient biological and mechanical function. A treatment may be valuable without achieving structural modification or true regeneration; however, describing symptomatic benefit as disease modification risks overstating the evidence (41, 50).

### 3.1 Surgical joint-preservation strategies

Surgical joint preservation may be appropriate in selected patients when correctable mechanical, meniscal, ligamentous, or focal osteochondral pathology contributes to symptoms or joint degeneration. These procedures aim to preserve native joint structures, improve biomechanics, protect cartilage and meniscal tissue, and maintain function while potentially delaying the need for arthroplasty. Patient selection and careful surgical planning are essential, particularly because outcomes depend on factors such as age and activity level, limb alignment, ligamentous stability, meniscal status, cartilage damage, and the extent of osteoarthritis. These procedures are generally most applicable to localized pathology rather than advanced diffuse or multicompartment degeneration (51, 52).

Meniscal preservation is an important component of joint-preserving surgery because the menisci contribute to load distribution, shock absorption, joint stability, lubrication, and proprioception (53). When technically feasible, repair is generally favored over meniscectomy to preserve native meniscal tissue and knee biomechanics (53, 54). Patient selection depends on factors including tear pattern and location, vascularity, chronicity, tissue quality, age, and activity level (54). A 2023 meta-analysis of 20 studies involving 31,783 patients found that meniscal repair was associated with lower rates of advanced osteoarthritis and progression to total knee arthroplasty compared with meniscectomy, although patient-reported outcomes and overall failure rates were not significantly different (53). Repair is not appropriate for all tears, and complex, degenerative, or poorly vascularized tears may be less suitable for repair and require alternative management (53, 54).

Anterior cruciate ligament reconstruction (ACLR) is an important joint-preserving procedure for patients with clinically significant instability, with the primary goals of restoring knee stability, reducing recurrent instability, and limiting secondary injury to the meniscus and articular cartilage (34). Although ACLR can improve mechanical stability and function, it does not reliably prevent post-traumatic osteoarthritis. A 2026 systematic review and meta-analysis of 18 studies including 33,540 patients found no significant difference in long-term radiographic osteoarthritis between ACLR and nonoperative management, with a mean follow-up of 12.3 years. Outcomes are influenced by concomitant meniscal and chondral injury, rehabilitation, graft integrity, and recurrent instability, emphasizing that reconstruction alone does not eliminate long-term degenerative risk (55).

Corrective osteotomy is an important joint-preserving option for patients with symptomatic unicompartmental disease associated with lower-limb malalignment. High tibial osteotomy (HTO) is most commonly used in younger, active patients with varus alignment and medial-compartment overload, whereas distal femoral osteotomy (DFO) is generally considered when the deformity originates from the femur, particularly in valgus knees with lateral-compartment disease. Proper patient selection requires assessment of alignment, compartment-specific cartilage and meniscal status, range of motion, activity level, and the extent of osteoarthritis, with advanced multicompartment degeneration representing an important limitation (52). In appropriately selected patients, HTO has been associated with return-to-sport or return-to-work rates of approximately 76%–87% and native-knee survival of about 70%–80% at 10 years, while reported 10-year survivorship after DFO ranges from approximately 65%–90% (52). Recent clinical data also demonstrate significant improvements in pain and patient-reported function following HTO or DFO, although reoperation, hardware irritation, hinge fracture, delayed or nonunion, and inaccurate correction remain important limitations (52, 56).

Osteochondral transplantation can restore both articular cartilage and subchondral bone in selected patients with symptomatic focal osteochondral defects. Osteochondral autograft transfer (OAT) is generally suited to younger, active patients with isolated small- to medium-sized lesions, commonly around 2–4 cm^2^, intact surrounding cartilage, and a stable knee. Mid- to long-term studies demonstrate sustained improvements in pain and function after OAT, although donor-site morbidity and the limited amount of available autograft tissue restrict treatment of larger defects (57). Osteochondral allograft transplantation (OCA) can address larger defects and avoids autograft donor-site morbidity, with reported graft survivorship of approximately 75%–80% at 10 years. However, graft availability, preservation and viability, osseous incorporation, and graft failure remain important limitations (58). Advanced or diffuse osteoarthritis is not an appropriate indication for OAT, and advanced osteoarthritis is a contraindication to OCA (57, 58).

Focal cartilage repair procedures are selected according to lesion size, location, patient characteristics, and the condition of the surrounding joint. Marrow-stimulation techniques such as microfracture are generally used for smaller symptomatic focal defects and can improve pain and function, although the fibrocartilage repair tissue may have limited durability over time. Autologous chondrocyte implantation and matrix-induced autologous chondrocyte implantation are more commonly considered for larger full-thickness defects, particularly in younger, active patients, and may provide sustained functional improvement. Scaffold-based strategies have expanded cartilage-restoration options but remain dependent on appropriate patient and lesion selection. Importantly, cartilage restoration should not be performed in isolation when substantial malalignment, meniscal deficiency, or ligamentous instability is present, because these mechanical factors can compromise outcomes (59).

Collectively, these procedures extend the joint-preservation continuum beyond symptom management by addressing structural and biomechanical contributors to degeneration. Their role remains greatest in appropriately selected patients with localized, correctable pathology before advanced diffuse osteoarthritis develops.

## 4 Foundational joint-preserving management

Exercise, education, and sustained weight management form the basis of KOA treatment. The American Academy of Orthopaedic Surgeons (AAOS) supports supervised, unsupervised, or aquatic exercise and weight loss in patients who are overweight or obese to improve pain and function (60). Obesity increases tibiofemoral loading and may also contribute to disease through adipokines and systemic low-grade inflammation (61). Weight-management strategies should extend beyond general advice to lose weight and may incorporate structured dietary modification, exercise, and behavioral or psychological support. A 2025 systematic review and network meta-analysis of 14 randomized trials involving 3,215 patients with KOA found that multiple weight-loss strategies produced greater weight reduction than control strategies. Meta-regression suggested that significant pain relief may be anticipated when patients achieve weight loss of approximately 7.4% or more (62). Education and self-management strategies can further support realistic goal setting, symptom management, self-efficacy, and sustained participation in lifestyle and rehabilitation programs (63).

Strengthening of the lower-extremity musculature, particularly the quadriceps and hip musculature, is an important component of KOA rehabilitation because improved muscular strength can enhance joint stability, shock absorption, gait, and functional capacity. Exercise programs may incorporate progressive resistance training, aerobic activity, aquatic exercise, flexibility and range-of-motion exercises, and neuromuscular training according to patient needs (63). A 2023 systematic review and network meta-analysis of 39 randomized controlled trials involving 2,646 patients found that multiple exercise modalities, including resistance training, stationary cycling, aquatic exercise, traditional exercise, and yoga, improved pain, stiffness, physical function, or quality of life in patients with KOA. Aquatic exercise may be particularly useful for patients who have difficulty tolerating land-based activity because buoyancy reduces weight bearing across the symptomatic joint (64).

Exercise prescription should be individualized and progressively adjusted according to disease severity, baseline functional capacity, comorbidities, symptoms, and patient goals. Physical therapy can incorporate progressive strengthening, range-of-motion exercises, balance and proprioceptive training, and gait retraining to improve mobility and neuromuscular control. Long-term benefit also depends on adherence, making patient education, realistic goal setting, and selection of activities that patients are willing and able to sustain important components of treatment. Psychological factors, including fear of movement and low self-efficacy, may limit participation; behavioral strategies and graded activity can therefore be incorporated to improve confidence, adherence, and continued engagement in rehabilitation (63).

Brace selection should be individualized according to lower-limb alignment, the predominantly affected knee compartment, and the presence of instability (65). Valgus or varus unloading braces may be considered for compartment-specific tibiofemoral OA to redistribute joint loading, whereas patellofemoral braces may be more appropriate when symptoms predominantly involve the patellofemoral joint. Hinged or stabilizing braces may also be considered when instability contributes to pain or functional limitation (65). Cane use may be considered when pain, instability, or impaired ambulation limits function, while other assistive devices should be selected according to individual mobility and stability needs (37).

NSAIDs administered orally or topically can reduce symptoms and facilitate activity and participation in rehabilitation when appropriate. Both the AAOS and ACR/Arthritis Foundation support topical NSAIDs for KOA, with the ACR/Arthritis Foundation recommending topical therapy before oral NSAIDs when feasible because topical administration results in lower systemic exposure (37, 60). Oral NSAIDs are also strongly recommended by both guidelines for improving pain and function when not contraindicated (37, 60). However, NSAIDs primarily provide symptomatic relief and have not been shown to preserve joint structure or modify the underlying progression of KOA (41). Their role within joint-preserving management is therefore indirect, through improved pain control, mobility, and participation in exercise and rehabilitation.

Pharmacologic treatment should be individualized according to gastrointestinal, cardiovascular, renal, and other comorbid risks. Oral NSAIDs are associated with gastrointestinal, cardiovascular, and renal adverse effects, supporting use of the lowest effective dose for the shortest necessary duration and careful patient selection and monitoring (66). Because many analgesics are available over the counter, patients should also be counseled regarding appropriate dosing and avoidance of medication duplication. Acetaminophen may be considered when NSAIDs are contraindicated or poorly tolerated, although guideline recommendations differ: AAOS recommends acetaminophen for improving pain and function when not contraindicated, whereas the ACR/Arthritis Foundation conditionally recommends short-term or episodic use because average treatment effects are small and regular use requires attention to hepatotoxicity (37, 60). The ACR/Arthritis Foundation also conditionally recommends duloxetine for KOA, particularly as an alternative or adjunct when conventional analgesic strategies provide inadequate relief, and conditionally recommends topical capsaicin for knee OA, recognizing its relatively modest treatment effect (37). Major clinical guidelines are broadly consistent regarding foundational management of KOA but differ in their recommendations for several pharmacologic and injectable therapies, as summarized in Table 1.

**Table 1:** Comparison of selected guideline recommendations for the nonoperative management of knee osteoarthritis.

Treatment	AAOS	ACR/AF	OARSI	ESCEO
Acetaminophen	Strong; if not contraindicated	Conditionally recommended	Conditionally not recommended	Weak; short-term rescue only
Duloxetine	Not addressed	Conditionally recommended	Conditional; widespread pain and/or depression	Weak; Step 3, especially for central sensitization
Hyaluronic acid	Not for routine use	Conditionally against	Conditionally recommended	Weak; if NSAIDs are unsuitable or inadequate
PRP	Limited; may improve pain/function	Strongly against	Strongly against	Not addressed
Oral NSAIDs	Strong; when appropriate	Strong; minimize dose/duration	Conditional; comorbidity- dependent	Strong; Step 2, risk-profile dependent
Topical NSAIDs	Strongly recommended	Strongly recommended	Strongly recommended	Strong; Step 1 add-on therapy

Recommendations are summarized from AAOS (60), ACR/AF (37), OARSI (38), and the 2019 ESCEO recommendations summarized by Arden et al. (39). Recommendation terminology is simplified for comparison; grading systems and treatment algorithms differ among organizations and are not directly equivalent.

Following knee injury, secondary prevention should focus on restoring strength and function, minimizing recurrent injury, preserving meniscal tissue when possible, and addressing clinically significant instability or malalignment to reduce continued abnormal mechanical loading of the joint (34, 53, 67, 68).

## 5 Intra-articular symptom-modifying therapies

### 5.1 Extended-release triamcinolone acetonide

Intra-articular corticosteroids and hyaluronic acid preparations encompass multiple formulations with differing pharmacologic properties and evidence bases. The following subsections discuss these major intra-articular symptom-modifying treatment classes, with TA-ER highlighted as a well-studied example of an extended-release corticosteroid formulation.

Intra-articular corticosteroids suppress synovial inflammation and can provide short-term symptomatic relief, with benefits generally greatest during the first several weeks after injection (69). Concern remains regarding repeated conventional injections: a two-year randomized trial of triamcinolone every three months found greater cartilage-volume loss without superior pain relief compared with saline (70). These findings should not be generalized to all corticosteroid formulations or injection strategies, but they underscore that symptomatic benefit and structural preservation are not synonymous outcomes.

Triamcinolone acetonide extended-release (TA-ER) embeds drug crystals in biodegradable poly(lactic-co-glycolic acid) microspheres. Pharmacokinetic studies demonstrated prolonged synovial residence and lower systemic exposure than standard crystalline suspension (71). In a phase III trial, a single 32-mg injection improved average daily pain (ADP) vs. saline at 12 weeks and improved several WOMAC and KOOS outcomes; however, ADP-based secondary comparisons with conventional triamcinolone were not statistically significant (72). A *post hoc* analysis of participants with unilateral KOA reported benefits lasting approximately five to six months, but the exploratory design and selected population limit generalization (73). TA-ER is FDA-approved for the management of osteoarthritis pain of the knee and is therefore best characterized as a longer-acting local symptom therapy rather than a demonstrated DMOAD (41, 73).

### 5.2 Hyaluronic acid viscosupplementation

Osteoarthritic synovial fluid has reduced viscoelasticity (74). Intra-articular hyaluronic acid (HA) viscosupplementation is intended to supplement the lubricating and shock-absorbing properties of synovial fluid (74, 75). Available HA preparations differ in molecular weight, cross-linking, concentration, and dosing schedule, contributing to heterogeneity across clinical studies and treatment responses (75). Meta-analysis suggests that HA injections may produce a gradual reduction in pain that peaks at approximately eight weeks and can persist for up to six months (76). Nevertheless, a large 2022 systematic review and meta-analysis concluded that, although viscosupplementation produced statistically significant improvements in pain and function, the magnitude of benefit was below established thresholds for clinical importance and was associated with an increased risk of serious adverse events. However, causal attribution of these serious adverse events to viscosupplementation remains uncertain (77). By contrast, a 2016 systematic review and network meta-analysis found that HA products were generally well tolerated, with transient local pain, swelling, and arthralgia representing the most common adverse reactions and very few treatment-related serious adverse events reported (78).

Given the mixed efficacy data and differing guideline recommendations summarized in Table 1 (37–39, 60), HA viscosupplementation may be considered selectively in patients with persistent symptoms despite foundational therapy and appropriate first-line pharmacologic treatment, particularly when other treatment options are ineffective, poorly tolerated, or contraindicated (39, 79). Patient comorbidities, disease characteristics, and patient preference should guide treatment decisions (79), while treatment cost and insurance coverage may also influence treatment selection and access (75). HA viscosupplementation is therefore best regarded as a symptom-modifying treatment rather than an established disease-modifying therapy (41, 75). Treatment should remain individualized according to patient characteristics, recognizing the heterogeneity of treatment response and uncertainty regarding long-term outcomes (75, 79).

## 6 Investigational mechanism-based, orthobiologic, and regenerative approaches

### 6.1 Cytokine-directed therapy

Although IL-1β is a potent inducer of cartilage catabolism in experimental models, clinical trials of IL-1–targeted therapies have not demonstrated consistent symptomatic or structural benefit in patients with OA (80). This disconnect may reflect OA heterogeneity and variable IL-1 biology (80–82), as well as limitations of intra-articular drug delivery, including rapid clearance from the synovial space (83). IL-6 is also implicated in synovitis, cartilage degradation, pain, and subchondral remodeling; soluble-receptor-mediated trans-signaling may amplify its effects across multiple cell types (84–87).

Gene therapy seeks sustained local protein expression after a single injection (88). Preclinically, PCRX-201 (formerly FX201) is a helper-dependent adenoviral vector encoding interleukin-1 receptor antagonist (IL-1Ra) under the control of an inflammation-responsive NF-*κ*B promoter. In a rat ACLT model, FX201 attenuated cartilage degeneration, subchondral bone remodeling, and synovial pathology, resulting in improved histologic OA outcomes (89). Clinically, an open-label phase I study involving 72 patients reported transient local knee reactions and exploratory improvements in pain, stiffness, and function, with fewer knee effusions observed following corticosteroid pretreatment (90). However, the trial lacked a randomized control group and was primarily designed to assess safety. Its clinical efficacy and potential structural effects remain unproven. PCRX-201 is currently being evaluated in a randomized, double-blind phase II trial (NCT06884865) in patients with painful KOA (91).

PPV-06 represents a different strategy based on active immunization intended to generate antibodies against IL-6. In a randomized, double-blind phase I study of 24 patients with inflammatory KOA, PPV-06 demonstrated acceptable short-term safety and immunogenicity without dose-limiting toxicity (92). The study was not powered to establish symptomatic or structural efficacy. PPV-06 has subsequently progressed to a registered phase II randomized, double-blind, placebo-controlled multicenter trial (NCT06605963) evaluating safety, pain, physical function, and structural outcomes in patients with inflammatory KOA (93). Both PCRX-201 and PPV-06 illustrate biologically targeted treatment, but neither is ready for routine clinical use (91, 93).

### 6.2 Cartilage restoration and tissue engineering

Cartilage repair and restoration strategies are important components of joint-preserving treatment for focal chondral and osteochondral defects of the knee. Among these approaches, MACI (autologous cultured chondrocytes on porcine collagen membrane) and Agili-C, a cell-free biphasic aragonite osteochondral scaffold, are designed to address anatomically defined defects rather than generalized whole-joint KOA (94, 95). Their clinical role therefore depends on careful patient selection, including defect characteristics, joint alignment, meniscal status, and the severity of underlying osteoarthritis.

MACI is indicated for the repair of single or multiple symptomatic full-thickness cartilage defects of the knee, with or without bone involvement, in adults. Severe knee osteoarthritis, defined in the product labeling as Kellgren–Lawrence grade 3 or 4, is a contraindication, and safety and effectiveness in patients older than 55 years have not been established (94). Long-term follow-up has demonstrated sustained clinical and MRI-based improvements in appropriately selected patients with focal cartilage defects, with favorable outcomes maintained at 10 years (96). Accordingly, MACI represents a joint-preservation strategy for focal cartilage pathology rather than a treatment for generalized whole-joint KOA.

Agili-C is a cell-free, biphasic aragonite osteochondral scaffold derived from purified coral exoskeleton and designed to support repair of cartilage and underlying subchondral bone. It is indicated for focal knee-joint surface lesions with a total treatable area of 1–7 cm^2^ in patients without severe osteoarthritis, corresponding to Kellgren–Lawrence grades 0–3 (95). In a multicenter randomized controlled trial, patients treated with the aragonite-based implant demonstrated greater improvement in overall KOOS and a lower treatment-failure rate at five years compared with the surgical standard of care consisting of arthroscopic debridement or microfracture. Favorable outcomes were also observed among patients with mild-to-moderate osteoarthritis (97). Nevertheless, Agili-C is intended to treat focal chondral or osteochondral lesions and should not be considered a treatment for generalized or end-stage whole-joint KOA.

Additional joint-preservation procedures may be selected according to defect size, subchondral bone involvement, and meniscal integrity (98, 99). Osteochondral autograft transplantation and osteochondral allograft transplantation provide cartilage and underlying subchondral bone for selected focal osteochondral defects (98). Meniscal preservation and repair are prioritized when feasible, while meniscal allograft transplantation may be considered in selected patients with symptomatic meniscal deficiency (99). In patients with clinically relevant malalignment and compartmental disease, alignment-correcting procedures such as high tibial or distal femoral osteotomy can redistribute joint loading and may be combined with cartilage or meniscal restoration procedures (100). These approaches further emphasize that successful joint preservation depends on correcting specific structural and biomechanical abnormalities rather than treating generalized whole-joint KOA.

Beyond established focal cartilage-restoration procedures, tissue engineering seeks to restore cartilage and osteochondral structure through combinations of cells, biomaterial scaffolds, and bioactive signals (101). Investigational approaches include mesenchymal stromal cells (MSCs), growth factors, hydrogels, three-dimensional bioprinting, and cartilage organoids. Although these technologies show promise for cartilage regeneration, disease modeling, and drug discovery, limitations in tissue integration, mechanical stability, cellular maturation, reproducibility, and clinical translation remain important challenges (101–103).

### 6.3 Photobiomodulation and platelet-rich plasma

Photobiomodulation (PBM) is a non-invasive therapy that uses red and near-infrared light to modulate cellular activity and inflammatory signaling (104). *In vitro*, near-infrared irradiation reduced IL-1β-induced expression of inflammatory cytokines (IL-1β, IL-6, and TNF-α) and matrix metalloproteinases (MMP-1 and MMP-3) in human chondrocytes (105), while more recent preclinical work implicates macrophage polarization and IL-6/JAK/STAT signaling (106). Higher-level evidence remains mixed. A 2015 systematic review and meta-analysis of nine randomized controlled trials involving 518 patients found no significant benefit of low-level laser therapy over placebo for pain or function (107), whereas a 2019 systematic review and meta-analysis of 22 placebo-controlled trials found significant reductions in pain and disability, particularly when wavelength-specific recommended doses were used (108). These discrepant findings may partly reflect substantial heterogeneity in PBM protocols, including differences in wavelength, energy density, power, treatment frequency, treatment site, and comparator (109). A 2025 double-blind randomized controlled trial found that photobiomodulation significantly reduced pain and improved WOMAC and KOOS outcomes compared with sham and control conditions in patients with knee osteoarthritis (110). However, structural preservation has not been established, and PBM is best considered an adjunctive nonpharmacological symptom-modifying modality (109).

Platelet-rich plasma (PRP) is an autologous blood-derived concentrate containing supraphysiologic platelet concentrations and associated growth factors involved in tissue repair and regeneration (111). A systematic review and meta-analysis of 18 randomized controlled trials found greater improvements in WOMAC scores with PRP than with hyaluronic acid (HA), and subgroup analysis showed significantly better Subjective IKDC scores with leukocyte-poor than with leukocyte-rich PRP (112). However, a five-year double-blind randomized controlled trial involving 192 patients with Kellgren–Lawrence grade 0–3 knee degeneration found that, although both PRP and HA improved symptoms and function, PRP did not provide superior overall clinical improvement or a significantly longer duration of symptomatic benefit (113).

Interpretation of the PRP literature is complicated by substantial variability in preparation methods, leukocyte content, platelet concentration and total platelet dose, activation method, fresh vs. stored preparations, number and interval of injections, patient age, body mass index, Kellgren–Lawrence grade, comparator treatment, HA formulation, and follow-up duration. The lack of standardized preparation and reporting further limits comparison across studies (111, 114, 115). A 2025 umbrella review of 17 systematic reviews and meta-analyses found that, although PRP generally appeared to provide greater symptomatic benefit than HA, the methodological quality of all 17 reviews was rated extremely low, and most evaluated outcomes were supported by evidence of low or extremely low quality (116). Furthermore, the RESTORE randomized clinical trial of 288 patients found no significant benefit of PRP over saline for either knee pain or MRI-measured medial tibial cartilage volume loss at 12 months, providing no evidence of structural disease modification in that study (117). Guideline recommendations for PRP also remain inconsistent, as summarized in Table 1. Consequently, despite evidence of symptomatic benefit in some studies, the clinical effectiveness of PRP in KOA remains under debate, and greater standardization of PRP products and treatment protocols is needed (114, 116).

### 6.4 Cell-based and other orthobiologic products

Cell-based products derived from bone marrow or adipose tissue are increasingly investigated for KOA, but the term “stem-cell therapy” encompasses heterogeneous preparations with different cell populations, processing methods, doses, and regulatory status (118, 119). MSCs may promote cartilage repair through a combination of differentiation, paracrine signaling, and immunomodulatory effects (119). Standardized manufacturing protocols, adequately powered randomized comparative trials, long-term safety surveillance, and clear regulatory oversight are required before these therapies can be incorporated into routine clinical care (118, 119).

## 7 Delaying arthroplasty: why preservation matters

Demand for primary and revision arthroplasty continues to increase internationally, with projections indicating sustained growth over the coming decades (120). TKA reliably improves pain and function for most patients, although recovery and patient-reported outcomes remain variable (121). Chronic pain affects a clinically important minority of patients (122), and dissatisfaction following TKA remains an important consideration, although contemporary estimates are lower than historical reports of approximately 20% (123). Registry evidence indicates that approximately 82% of TKAs remain unrevised at 25 years; this reflects implant survivorship, not proof of normal function (124). Younger patients have demonstrated higher early revision rates than older patients, with additional evidence of greater reoperation and rerevision risk following revision TKA (125, 126).

These limitations support effective joint-preservation strategies, particularly for younger or higher-demand patients; however, delaying arthroplasty is not inherently beneficial when pain and functional limitation remain disabling. The objective is timely escalation: preserve native tissue and function when realistic, while using arthroplasty when its expected benefit exceeds that of continued nonoperative or restorative care (127).

## 8 Future directions: precision and disease modification

Precision KOA care will require improved identification and stratification of clinical phenotypes and molecular endotypes to better match patients with targeted therapies (6, 128). Clinical assessment and imaging can help identify structural abnormalities and major pain drivers, including inflammatory, mechanical, and bone-marrow-lesion-related phenotypes (6). Transcriptomic and other “omics” approaches may further identify molecular subgroups and enable deep phenotyping of KOA, but tissue acquisition, sample quality, data interpretation, and bioinformatic complexity remain important challenges (129).

Biomarkers must also be classified by purpose. Diagnostic biomarkers identify disease; prognostic biomarkers estimate progression; predictive biomarkers identify likely response to a specific treatment; and pharmacodynamic biomarkers demonstrate biological target engagement. Current candidates include collagen-turnover products, inflammatory mediators, metabolites, and microRNAs (130, 131). Despite substantial progress, most remain research tools rather than validated clinical decision tests.

Representative DMOADs were selected to highlight different biological targets and contrasting clinical trial outcomes rather than provide an exhaustive review of the pipeline. Therapeutic development must be interpreted cautiously. Lorecivivint, an intra-articular CLK2/DYRK1A inhibitor and Wnt-pathway modulator, did not meet the primary 12-week pain endpoint in the phase III OA-11 trial, with no clear treatment effects across other major symptomatic or structural outcomes (132). In contrast, results reported in a conference abstract from the OA-07 extension study suggested more favorable outcomes with repeat lorecivivint injections, including improvements in pain and a signal of less medial joint-space-width loss over longer follow-up; however, interpretation is limited by the abstract-only report, extension design, and placebo crossover (133). In January 2026, Biosplice announced submission of a New Drug Application (NDA) to the FDA for lorecivivint; however, NDA submission does not constitute regulatory approval or confirm disease-modifying efficacy (134). Sprifermin, a recombinant human fibroblast growth factor 18, illustrates a different challenge: discordance between structural and symptomatic outcomes. In the FORWARD trial, it demonstrated a dose-dependent increase in femorotibial cartilage thickness, with the structural effect maintained through five years (135). However, WOMAC pain improved substantially across all treatment groups, including placebo, without clear symptomatic superiority for sprifermin in the overall study population (135).

By contrast, the ADAMTS-5 inhibitor S201086/GLPG1972 failed to significantly reduce cartilage loss or improve symptoms compared with placebo in the phase II ROCCELLA trial (136). Similar lessons apply to DMOAD development: mechanistic plausibility and subgroup signals do not substitute for consistent achievement of primary endpoints, meaningful structural benefits, and demonstrated long-term safety. Future progress will likely require earlier intervention, enriched trial populations, integrated clinical and molecular stratification, and outcome measures that link tissue preservation to improvements that matter to patients (137).

Trial design should also account for the slow pace of structural change, fluctuating symptoms, contextual responses to intra-articular interventions, and competing pain mechanisms. Adaptive trial designs may improve trial efficiency (138), while biomarker-enriched enrollment strategies may increase the likelihood of detecting treatment effects; however, subgroup findings require prospective validation (137). Ultimately, precision medicine will be clinically meaningful only if diagnostic or molecular stratification changes treatment selection and results in superior patient outcomes compared with conventional clinical assessment alone.

## 9 Discussion

Across the treatment continuum reviewed, the most consistent high-level clinical evidence supports foundational management aimed at improving pain, function, physical capacity, and modifiable risk factors. Exercise, education, weight management when appropriate, and established pharmacologic therapies such as topical or oral NSAIDs remain central components of KOA care and are broadly supported by major clinical guidelines (37, 38, 60). In contrast, the evidence supporting intra-articular and orthobiologic interventions is more heterogeneous. Intra-articular corticosteroids can provide short-term symptomatic relief (69), whereas the magnitude and clinical relevance of benefit from hyaluronic acid remain debated across systematic reviews and guidelines (75, 77, 79). PRP has demonstrated symptomatic improvement in randomized trials and meta-analyses, including comparisons with hyaluronic acid (112, 116), but substantial variability in product preparation, treatment protocols, patient populations, and study quality limits definitive conclusions (114, 116). Thus, greater biological sophistication of an intervention should not be interpreted as indicating a correspondingly higher level of clinical support.

An important distinction across the therapies reviewed is that symptomatic improvement should not be interpreted as evidence of structural disease modification or regeneration (49). Intra-articular corticosteroids, hyaluronic acid, photobiomodulation, and PRP are primarily supported as symptom-modifying interventions, with limited or inconsistent evidence that they alter the structural progression of generalized KOA (70, 74, 109, 117). Conversely, structural improvement does not necessarily translate into clinically meaningful symptomatic benefit. Sprifermin increased femorotibial cartilage thickness in the FORWARD trial yet did not demonstrate clear symptomatic superiority over placebo in the overall study population (135). Cartilage-restoration procedures such as MACI and Agili-C can produce structural and clinical improvements in appropriately selected focal defects (94–97), but these findings should not be extrapolated to regeneration of the diffusely osteoarthritic joint. Accordingly, claims of clinically meaningful disease modification are strongest when durable structural benefit is accompanied by clinically meaningful improvements in pain, function, or quality of life rather than relying on imaging or other surrogate outcomes alone (49, 137).

Many of the most biologically targeted approaches remain preclinical or in early-phase clinical development, underscoring the gap between mechanistic plausibility and established therapeutic efficacy. Senolytic and senomorphic strategies, tissue-engineering technologies, organoids, and several cell-based approaches remain predominantly investigational (17, 101–103, 118, 119), whereas targeted therapies such as PCRX-201 and PPV-06 have progressed into early clinical trials but have not yet demonstrated definitive symptomatic or structural efficacy (90–93).

Patient selection is likely to be a major determinant of future success because KOA encompasses heterogeneous mechanical, inflammatory, metabolic, structural, and pain-related phenotypes that may respond differently to the same intervention (6, 7, 128). Biomarkers, imaging characteristics, transcriptomic profiles, and molecular endotypes may eventually help enrich clinical trials and guide treatment selection, but most candidate biomarkers remain research tools rather than validated clinical decision tests (128–131). Precision treatment therefore remains promising, but biomarker-guided therapy is not yet ready for routine clinical use.

Translation of available evidence into routine clinical practice is further complicated by disagreement among major clinical guidelines and by practical implementation barriers. Recommendations are broadly aligned for foundational interventions such as exercise and NSAID therapy but diverge substantially for treatments including hyaluronic acid and PRP, reflecting differences in evidence grading, thresholds for clinically meaningful benefit, study populations, and interpretation of heterogeneous trial data (37, 38, 60). Treatment selection may also be influenced by cost, insurance coverage, availability of specialized procedures, clinician expertise, and patient preferences (37, 79, 119). These challenges are particularly relevant for orthobiologic and cell-based products, for which differences in preparation methods, manufacturing standards, terminology, and regulatory status can limit comparability between studies and countries (114, 118, 119). Consequently, clinical adoption should consider not only statistical significance and reported efficacy but also reproducibility, accessibility, regulatory oversight, treatment burden, and whether observed improvements are meaningful to patients in routine practice (49, 119, 137).

## 10 Limitations

This review has several limitations. First, it was conducted as a narrative rather than a systematic review and therefore may be subject to study-selection bias despite prioritizing higher-level and clinically relevant evidence where available. Second, the evidence base for KOA treatment is rapidly evolving, particularly for DMOADs, orthobiologics, gene-based therapies, and precision-medicine approaches, and emerging findings may alter current interpretations. Comparisons across interventions are also limited by substantial heterogeneity in patient populations, disease severity, treatment protocols, comparators, follow-up duration, and definitions of symptomatic and structural outcomes. Direct head-to-head evidence between many treatment strategies remains limited. In addition, products such as PRP, cell-based therapies, and other orthobiologics may differ considerably in preparation, composition, manufacturing standards, and regulatory status, including across countries. These factors limit direct comparison and should be considered when interpreting the generalizability and clinical applicability of the evidence reviewed.

## 11 Conclusion

Foundational care remains central to KOA management, with exercise, education, weight management when appropriate, and established symptom-directed therapies providing the basis for improving pain, function, and physical capacity. Joint-preservation strategies should remain individualized according to disease stage, structural abnormalities, symptoms, and patient priorities.

Symptomatic improvement should not be equated with structural disease modification or regeneration. Although emerging biologic, molecular, gene-based, and tissue-engineering approaches have identified potential therapeutic pathways, evidence of durable structural modification accompanied by clinically meaningful patient benefit remains limited. Advances in phenotyping, imaging, biomarkers, transcriptomics, and molecular endotyping may ultimately enable more precise treatment selection; however, biomarker-guided therapy is not yet ready for routine clinical use.
